# Invasive meningococcal disease in three siblings with hereditary deficiency of the 8^th^ component of complement: evidence for the importance of an early diagnosis

**DOI:** 10.1186/s13023-016-0448-5

**Published:** 2016-05-17

**Authors:** Rosa Maria Dellepiane, Laura Dell’Era, Paola Pavesi, Paolo Macor, Mara Giordano, Luca De Maso, Maria Cristina Pietrogrande, Massimo Cugno

**Affiliations:** Department of Pediatrics, Fondazione IRCCS Cà Granda Ospedale Maggiore Policlinico, University of Milan, Milan, Italy; Department of Life Sciences, University of Trieste, Trieste, Italy; Department of Health Sciences, Laboratory of Genetics, University of Eastern Piedmont and Interdisciplinary Research Center of Autoimmune Diseases, Novara, Italy; Department of Pathophysiology and Transplantation, Internal Medicine, University of Milan, Fondazione IRCCS Ca’ Granda, Ospedale Maggiore Policlinico, Milan, Italy

**Keywords:** Complement deficiency, C8 deficiency, Meningococcal disease, *Neisseria meningitidis*

## Abstract

**Background:**

Deficiency of the eighth component of complement (C8) is a very rare primary immunodeficiency, associated with invasive, recurrent infections mainly caused by Neisseria species. We report functional and immunochemical C8 deficiency diagnosed in three Albanian siblings who presented with severe meningococcal infections at the age of 15 years, 4 years and 17 months, respectively. The youngest suffered serious complications (necrosis of fingers and toes requiring amputation).

**Methods:**

Functional activity of the classical, alternative and mannose-binding lectin complement pathways was measured in serum from the 3 siblings and their parents (37-year-old woman and 42-year-old man). Forty healthy subjects (20 males and 20 females aged 4–38 years) served as normal controls. Serum complement factors were measured by haemolytic assays and immunoblotting. Sequence DNA analysis of the C8B gene was performed.

**Results:**

Analyses of the three complement pathways revealed no haemolytic activity and also absence of C8beta in serum samples from all three siblings. The genetic analysis showed that the three siblings were homozygous for the p.Arg428* mutation in the C8B gene on chromosome 1p32 (MIM 120960). The parents were heterozygous for the mutation and presented normal complement activities. A 2-year follow-up revealed no further infective episodes in the siblings after antibiotic prophylaxis and meningococcal vaccination.

**Conclusions:**

Complement deficiencies are rare and their occurrence is often underestimated. In presence of invasive meningococcal infection, we highlight the importance of complement screening in patients and their relatives in order to discover any genetic defects which would render necessary prophylaxis to prevent recurrent infections and severe complications.

## Background

The terminal complement pathway comprises five proteins which become combined together to form the membrane attack complex (MAC) [1]. This is a major effector mechanism of humoral immunity; however the MAC cannot form if any of components are absent and affected patients have liability to bacterial infections including *Neisseria meningitidis* infections.

In particular, the 8^th^ complement component (C8), together with C5, C6, C7 and C9, assemble on bacterial membranes to form the lethal pore-like MAC. C8 is composed of three subunits (alpha, beta and gamma) which are encoded by three separate genes (C8A, C8B and C8G). Complement deficiencies represent approximately 1–6 % of all primary immunodeficiencies but this may go up to 10 % in certain communities [2–6]. In particular a recent study had shown that C8 deficiency represents 8 % of complement deficiencies across Europe [5]. The prevalence of congenital complement deficiency has been calculated to be about 0.03 % in the general European population, excluding MBL (mannose binding lectin) deficiency which has been estimated to occur in its homozygous form in about 5 % of the population [2, 7]. Inherited deficiencies of terminal complement components result in increased susceptibility to infections, particularly Neisseria species. However heterozygous carriers are not susceptible to these infections [8].

Unlike in the general population, where the infections mainly affect children in the first years of life, in patients with C8 complement deficiency the average age of onset was found to be 17 years and only 10 % of the cases occurred before 5 years of age. Recurrent disease occurred in 45 % [9]. Furthermore, some of these patients presented a milder course of the disease with a 5 to 10 fold decrease in the probability of death, when compared to meningococcal disease (MD) in the general population [9].

Here, we report a long history of invasive meningococcal disease in three C8-deficient Albanian siblings and severe complications in the youngest sister. The clinical course was favourable after patients had received meningococcal vaccination and antibiotic prophylaxis had been started.

## Methods

### Patients

Three patients attended the University Hospital in 2013 for evaluation of suspected immunodeficiency. They were Albanian siblings, a boy (16 years old) and two girls (14 and 6 years old respectively) and they presented with a long history of MD. Their parents, a 37-year-old woman and a 42-year-old man, were apparently healthy.

All three siblings were subjected to in-depth investigations to rule out primary immunodeficiencies. Tests showed normal values of serum immunoglobulins, IgG subclasses, and T and B cells numbers and activity. Functional activities of the classical and alternative pathways of complement were measured. Asplenia was excluded by abdominal ultrasound.

Forty healthy subjects (20 males and 20 females aged 4–38 years) served as normal controls for the complement studies.

### Eligibility

The study was conducted following the ethical principles of the Declaration of Helsinki, regulatory requirements and the code of Good Clinical Practice. The parents of the 3 patients gave their written informed consent for genetic studies.

### Functional tests for the classical, alternative and mannose-binding lectin complement pathways

These tests were performed in accordance with the manufacturer’s instructions (Wieslab Complement System; Euro-Diagnostica, Malmö, Sweden) through the three complement pathways with the terminal complement complex C5b-9 used as the common detection system [10]. The wells of the microtiter strips were coated with specific activators of the classical, alternative or mannose-binding lectin pathways. The serum samples were diluted in buffer containing specific blockers in order to ensure that only one pathway was activated during incubation. The wells were then washed, and C5b-9 was detected with a specific alkaline phosphatase-labelled antibody against the neoantigen expressed on C9 during C5b-9 formation. After a further washing step, the specific antibodies were detected by means of incubation with the alkaline phosphatase substrate solution. As the amount of complement activation correlates with colour intensity and is measured in terms of absorbance, the results were expressed as percentages of the activity of a standard sample (i.e. normal pooled serum fixed at 100 %).

### Haemolytic assay

This assay was done as previously described [11]. Briefly, antibody-sensitized sheep erythrocytes (EA) were prepared with sub-agglutinating amount of rabbit IgM antibodies. Haemolytic activity was evaluated by mixing dilutions of test sera in glucose veronal-buffered saline (GVBS) with 50 μl of 1 % EAC1-3b (i.e. EA incubated with a C5 deficient serum to form C3 convertase [C1-3b]) to a final volume of 250 μl. After incubation at 37 °C for 30 min, red cell lysis was calculated by measuring the OD_415_. Haemolytic activity was expressed as a percentage of lysis induced by water.

### Sodium dodecyl sulphate-polyAcrylamide gel electrophoresis (SDS-PAGE) and immunoblotting analysis

Serum samples were subjected to SDS-PAGE on a 10 % gel under non-reducing conditions followed by electrophoretic transfer onto nitrocellulose membrane (Hybond ECL, Amersham, Milano, Italy) using the semidry Semiphor transfer unit (Heifer Scientific Instruments, San Francisco, CA). The membranes were incubated with 1/1000 goat IgG anti-C8 (Quidel, San Diego, CA, USA) for 1 h at 37 °C followed by 1/2,000 AP-conjugated anti-goat Ig (Sigma–Aldrich) for 1 h at 37 °C. The enzymatic reaction was developed as previously described [12]

### Enzyme-linked immunosorbent assay (ELISA) for C8

C8 was measured in serum samples by a sandwich ELISA using goat IgG anti-C8 to bind C8 and the same polyclonal antibody labeled with biotin to reveal bound C8 followed by AP-conjugated streptavidin (Sigma–Aldrich). The enzymatic reaction was developed using PNPP (Sigma–Aldrich) as previously described [13].

### Genetic analysis

Genomic DNA was amplified by PCR using primers designed to amplify the coding region and the intron/exon boundaries of the 12 exons of the C8B gene. PCR has been performed using the conditions and primers reported by Arnold et al. [14].

The PCR products were visualized on a 2 % agarose gel and purified using ExoSAP-IT enzymatic PCR clean up system (Affymetrix, Santa Clara CA). The purified products were then sequenced with the Big Dye Terminator kit (Applied Biosystems, Foster City, CA) and analysed on an ABI PRISM 3100 Genetic Analyzer (Applied Biosystems, Foster City, CA).

## Results

### Clinical features

The first patient (patient 1) is a 16-year-old male with a clinical history of sepsis due to nongroupable *Neisseria meningitides* (capsule null locus strain – serotype 25) at the age of 15 years. The clinical course was favourable and he was discharged in good clinical conditions after 10 days of intravenous antibiotic therapy. His two sisters had a history of previous meningococcal sepsis episodes also due to *Neisseria meningitidis*. The first sister (patient 2), aged 14, had suffered from MD at the age of 4 years (10 years before), while the younger sister (patient 3), aged 6 years old, had suffered from sepsis due to *Neisseria meningitidis* (serogroup B-serotype 14) at 17 months of age (5 years before); this was complicated by necrosis of fingers and toes which required amputation. The parents were not related to each other and did not have histories of severe infections.

### Laboratory findings

Complement analyses revealed undetectable functional activity of the classical, alternative and lectin pathways in all the three patients. Complement activity in the parents was normal for all the three pathways (Table 1). Further analyses were performed after recovery. The test of complement activity showed C8 deficiency in all siblings. These data were obtained with a haemolytic assay and adding sera from patients with previously characterized single complement component deficiencies. There were a clear and specific absence of the beta chains of C8 in siblings’ sera; the activities were restored by adding purified human C8 in the same lytic test (Table 2).

**Table 1:** Activity of the three complement pathways in the three patients and their parents

	Classical pathway	Alternative pathway	Lectin pathway
	%	%	%
Patient 1	0	0	0
Patient 2	3	0	0
Patient 3	3	0	0
Mother	116	92	120
Father	122	91	125
Normal range	69–129	30–113	0–125

**Table 2:** Evaluation of complement deficiency by haemolytic assay in serum from the 3 patients after adding serum with the deficiency (def) of a single complement component

Patient’s serum + :	Patient 1	Patient 2	Patient 3
	(% of lysis)	(% of lysis)	(% of lysis)
C5 def serum	99	95	90
C6 def serum	94	95	79
C7 def serum	100	100	96
C8 alpha-gamma def serum	95	87	88
C8 beta def serum	0	0	0
C8 beta def serum + human C8	99	100	94
C9 def serum	100	100	100

**Fig. 1:**
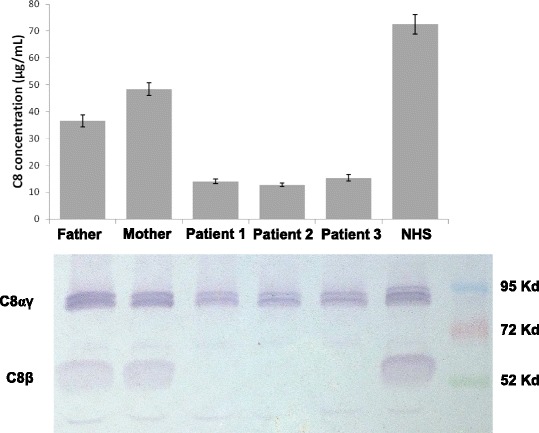
*Upper panel*. Levels of C8 antigen in serum from the three patients and their parents as well as in normal human serum (NHS). *Lower panel*. Sodium Dodecyl Sulphate-PolyAcrylamide Gel Electrophoresis (SDS-PAGE) and immunoblotting analysis of C8 in serum from the three patients and their parents as well as in NHS. The last lane on the right refers to molecular markers of known molecular weight

Moreover the deficiency of the C8 beta subunit in these patients was confirmed by western blot using anti-C8 antibody. The lane of C8 beta in the patient’s sera was absent while in the parents’ sera was present but reduced in amount (Fig. 1, lower panel). In the three patients, serum levels of C8 antigen, tested on 5 dilutions, resulted 13.00 ± 0.64 μg/ml, 12.00 ± 0.86 μg/ml and 15.00 ± 1.17 μg/ml, respectively, whereas in the parents, C8 serum levels were 52.00 ± 2.29 μg/ml (mother) and 34.00 ± 2.24 μg/ml (father). In normal human plasma, C8 serum levels were 72.50 ± 3.54 μg/ml (Fig. 1, upper panel).

**Fig. 2:**
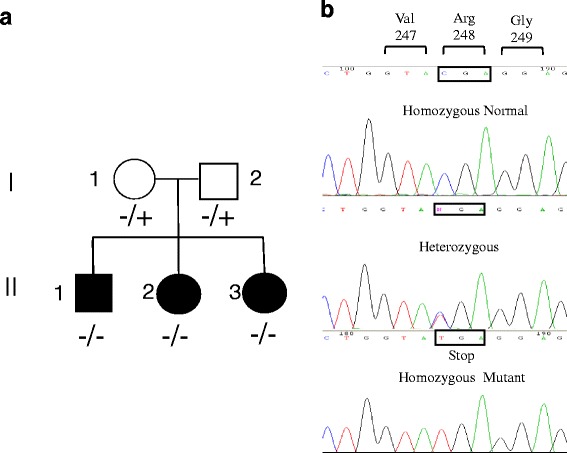
**a** Pedigree of the family. Filled symbols indicate affected individuals. The genotype for each family member is reported: +/− indicated the heterozygous and −/− the homozygous for the mutant allele. **b** Direct fluorescent sequencing of the exon 9 of the C8B gene. A control sequence homozygous for p.248Arg is reported along with the sequences from a heterozygous parent and a sib homozygous for the p.248Stop

Sequence analyses of exon 1–12 of the C8B gene on chromosome 1p32 in the 3 siblings revealed the presence of the nucleotide substitution, c.1282C>T that introduces a premature stop codon (p.Arg428*.) in exon 9 responsible for most reported cases of C8 beta deficiency (MIM 120960) [15]. The parents were heterozygous for the same mutation (Fig. 2).

### Treatment and course

After the diagnosis has been made the patients were immunized with the quadrivalent meningococcal conjugate vaccine (Menveo®, MenACWY-CRM; Novartis Vaccines and Diagnostics S.r.l., Siena, Italy) and as soon as possible with the vaccine against *N. meningitides* serogroup B (Bexero®, Novartis Vaccines and Diagnostics S.r.l., Siena, Italy). Antibiotic prophylaxis with amoxicillin was also started. They had no further episodes in the last two years.

## Discussion

The deficiency of C8 may be due to the lack of the beta subunit or to the lack of alpha and gamma subunits which are linked. The deficiency of C8 beta is more frequent in the Caucasians, while the lack of alpha-gamma chain is more frequent in Asian and African populations [15]. The three affected siblings were homozygous for the most commonly reported C8 beta mutation, namely c.1282C>T leading to a premature stop codon (p.Arg428*). This accounts for 85 % of mutations reported as causing C8 beta deficiency [15]. Laboratory data in the three patients and their parents are in agreement with the genetic profile and we observed absence of C8 activity and C8 beta subunit in the three patients and normal C8 activity with reduced levels of C8 antigen and C8 beta subunit in their parents. In the three patients, the absence of C8 activity is due to the absence of the beta subunit while the low levels of C8 antigen are due to the presence of the alpha and gamma subunits. In their parents, even if C8 antigen levels are about half of the normal, C8 activity is normal.

Deficiencies of complement components are rare diseases and their diagnosis is often underestimated. The meningococcal infection affects 40 % of individuals with late components complement deficiencies and MD may be the first manifestation of complement deficiency [16] with mean age of onset 17 years; only 10 % of the cases occur before 5 years of age [7]. Meningococcal infection in two of our patients has occurred before 5 years, at 17 months and 4 years of age respectively. The delay in diagnosis can lead the patient to develop recurrent meningococcal infections; in the literature it is known that another meningococcal infection occurs in 45 % of the patients [17]. Although meningococcal infections in patients with deficiency of complement are generally not lethal, the younger sister suffered serious complications (necrosis of fingers and toes that required amputation). These severe complications could have been avoided if the correct diagnosis had been made after the first episode of meningococcal infection in the sister.

Testing all MD patients for complement function or their DNA for selected gene defects known to circulate in the area is an option [6]. Although serious sequelae can develop because of any episode of meningococcal infection it has been observed that patients who have suffered recurrent MD infections become the worst affected and can have the most long term sequelae [7] and long term prophylaxis with penicillin was used to protect patients from further episodes. A recent study revealed that there is no agreement about of antibiotic prophylaxis in these patients [5]. However previous studies [18] demonstrated that meningococcal vaccination does not eliminate the risk of meningococcal disease and that in the individuals with inherited deficiencies of terminal complement components, meningococcal infection frequently involves unusual rare serotypes [9, 19, 20]. In our patient 1, capsule null locus strain, generally not pathogen, was found responsible for MD. For these reasons we decided to prescribe prophylactic amoxicillin to all three siblings. The lack of infective episodes during a 2-year follow-up further supports our view.

## Conclusion

This long family history highlights the importance of early diagnosis and indicates the need for complement evaluations in cases of invasive MD involving more than one family member. As reported by Hoare et al. [21] the presence of complement deficiency in children with MD is rare event that doesn’t always warrant further investigations. Indications to screen for complement deficiencies in patients with MD are, beyond family history as in our case, repeated neisserial infections, infection with unusual *Neisseria* serogroup, fulminant disease in males, unusual course of the illness, coexisting angio-oedema and autoimmune or connective tissue disorders [21]. The diagnosis of complement defects allows not only the prevention of recurrent infections by antibiotics and specific immunization of the patient, but also the disclosure, through appropriate genetic counselling, of other affected family members in order to prevent infections by antibiotic prophylaxis and immunization also in them.

### Ethics approval and consent to participate

The study was conducted following the ethical principles of the Declaration of Helsinki, regulatory requirements and the code of Good Clinical Practice. The parents of the 3 patients gave their written informed consent for genetic studies.

### Consent for publication

The parents of the three paediatric patients gave their written informed consent to publish their own data and the data of their children.

### Availability of data and supporting materials section

All the data used are presented in the manuscript.

## Abbreviations

C8: 8 ^th^ complement component
MAC: Membrane attack complex
MBL: Mannose binding lectin
EA: Antibody-sensitized sheep erythrocytes
GVBS: Glucose veronal-buffered saline
MD: Meningococcal disease
SDS-PAGE: Sodium dodecyl sulphate-polyacrylamide gel electrophoresis
ELISA: Enzyme-linked immunosorbent assay
